# Conformational and Supramolecular Aspects in Chirality of Flexible Camphor-Containing Schiff Base as an Inducer of Helical Liquid Crystals

**DOI:** 10.3390/molecules28052388

**Published:** 2023-03-05

**Authors:** Vladimir Burmistrov, Alena Batrakova, Viktor Aleksandriiskii, Igor Novikov, Konstantin Belov, Ilya Khodov, Oskar Koifman

**Affiliations:** 1 Research Institute of Macroheterocycles, Ivanovo State University of Chemistry and Technology, Sheremetev Av. 7, 153000 Ivanovo, Russia; 2 Institute of Solution Chemistry, Russian Academy of Sciences, Akademicheskaya St. 1, 153045 Ivanovo, Russia

**Keywords:** camphor-containing Schiff base, conformation, association, chirality, liquid crystals, inducer, helical twisting power

## Abstract

The experimental and theoretical study of influence of the conformational state and association on the chirality of the stereochemically nonrigid biologically active bis-camphorolidenpropylenediamine (CPDA) and its ability to induce the helical mesophase of alkoxycyanobiphenyls liquid–crystalline binary mixture was carried out. On the basis of quantum-chemical simulation of the CPDA structure, four relatively stable conformers were detected. A comparison of the calculated and experimental electronic circular dichroism (ECD) and ^1^H, ^13^C, ^15^N NMR spectra, as well as specific optical rotation and dipole moments, allowed to establish the most probable trans-gauche conformational state (tg) of dicamphorodiimine and CPDA dimer with a predominantly mutually parallel arrangement of molecular dipoles. The induction of helical phases in LC mixtures based on cyanobiphenyls and bis-camphorolidenpropylenediamine was studied by polarization microscopy. The clearance temperatures and the helix pitch of the mesophases were measured. The helical twisting power (HTP) was calculated. The decrease in HTP with increasing dopant concentration was shown to be connected with the CPDA association process in the LC phase. The effect of camphor-containing chiral dopants of various structures on nematic LCs was compared. The values of the permittivity and birefringence components of the CPDA solutions in CB-2 were measured experimentally. A strong effect of this dopant on the anisotropic physical properties of the induced chiral nematic was established. A significant decrease in the dielectric anisotropy was associated with the 3D compensation of the LC dipoles during the formation of the helix.

## 1. Introduction

Azomethines or Schiff bases are widely used and diverse organic synthetic compounds. Chiral azomethines are widely used in the pharmaceutical industry for the production of drugs with antibacterial [1], antifungal [2] and antioxidant activity [3]. Schiff bases are promising ligands [4,5] for the synthesis of complexes with catalytic properties, sensors [6] and can exhibit liquid crystal properties [7].

Biologically active azomethines based on natural terpenoids, such as camphor and borneol are of particular interest due to their high availability, enantiomeric purity, optical activity and low toxicity [8,9]. Camphor-based Schiff bases are effective catalysts for a variety of reactions in asymmetric syntheses [10,11,12,13]. The ability to photoisomerize camphoroquinone imines allows them to be used as the basis for molecular switches [14]. Camphorquinone derivatives are promising as photoinitiators for biomedical applications [15]. Thermal inversion of nitrogen and direct photoinduced rotation around the C=N bond in camphorquinone imines ensures their use as molecular motors [16]. However, the greatest attention is drawn to the biological activity of azo derivatives of such terpenoids as camphor and borneol [17]. Camphorimines have a strong inhibitory activity against influenza viruses, while the 1,7,7-trimethylbicyclo [2.2.1]heptan-2ylidene group is responsible for antiviral activity. Triazole derivatives of camphor are able to inhibit the reproduction of smallpox viruses [18], and camphor sulfamide derivatives are effective in the treatment of inflammatory diseases by blocking the chemokine receptor [19]. Biological activity is also exhibited by camphorimine complexes of metals, in particular Ag(I), which have antibacterial and antifungal effects [20].

When developing pharmacological preparations, knowledge about the influence of structure on their biological activity is of particular relevance [21]. Thus, when analyzing the antiviral activity of camphoromonoimines and camphecenes with aliphatic substituents, it was shown that compounds with a short alkyl chain have the greatest virus-inhibiting effect [22,23]. At the same time, for symmetric camphorodiimines, the compound with the –(CH_2_)_6^−^_ linker shows the highest inhibitory activity against the influenza virus, and the lowest toxicity with –(CH_2_)_12^−^_ [24]. In addition, the stereochemistry of camphor derivatives can also affect their biological activity, as was shown in the case of mono and bi-camphoroacylhydrazones [25].

Enantiomeric purity, optical activity, and the absence of absorption in the visible region of the spectrum of terpene derivatives allow them to be considered as promising chiral inductors of helical liquid–crystal phases, which have a number of advantages and additional capabilities compared to uniaxial nematic LCs. In [26], 1R(+)1,7,7-trimethylbicyclo [2,2,1]heptane-[2,3,b]-2,3-dicyanopyrazine (R(+)CDCP) with optical rotation +22.4° [27] was studied as the chiral dopant for the mixture of polar nematic cyanobiphenyls. It was shown that the polar dopant R(+)CDCP induces a chiral nematic phase and has a strong effect on its dielectric anisotropy due to the supramolecular compensation of dipoles in the helical structure of polar LCs.

Dinitrile R(+)CDCP was used to obtain a symmetrical macroheterocycle, dicamphor-containing hemiporphyrazine, as an inducer of a helical LC phase consisting of a six-component mixture of cyanobiphenyls. Cross-condensation of R(+)CDCP was shown to lead to an increase in specific rotation of up to +88° and a four-fold increase in the efficiency of chiral induction (helical twisting power) [28]. In this case, anomalous temperature dependences of the permittivity and viscosity of the LC composition are observed due to the competing action of the dopant, which twists the mesophase and the magnetic field or LC flow, which tend to unwind the helix.

**Scheme 1:**
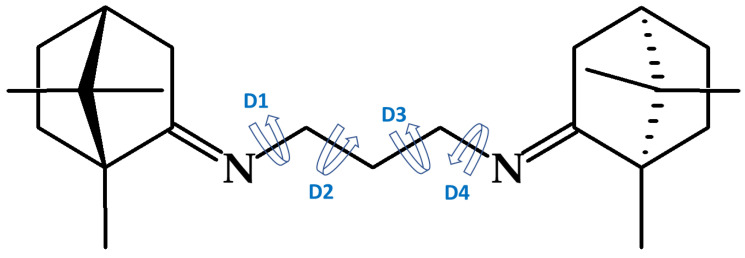
Biscamphorolidenpropylenediamine {(1*R*,2*E*,4*R*)-1,7,7-trimethyl-*N*-(3-{[(1*R*,2*E*,4*R*)-1,7,7-trimethylbicyclo-[2.2.1]heptan-2-ylidene]amino}propyl)bicyclo [2.2.1]heptan-2-imine} (CPDA).

The chiral camphor-containing dopants mentioned above have cyclic or macrocyclic structures that significantly limit their conformational mobility. The structurally non-rigid derivatives of terpenoids with aliphatic linkers described above are of interest, especially when they are in an orientationally ordered liquid crystal matrix. In this regard, this work is devoted to study the effect of the conformational and associative state of bis-camphorolidenpropylenediamine (CPDA, Scheme 1) on its chirality and induction of the helical mesophase based on nematic LC.

## 2. Results and Discussion

Due to the high rate of conformational transitions of bis-camphorolidenalkylenediamines flexible aliphatic linkers and the impossibility of separating and isolating relatively stable rotamers, the main initial method for studying their structure in the gas or liquid phase is quantum chemical simulation [29]. At the same time, the adequacy assessment of the calculation results can be carried out exclusively by independent experimental methods (spectroscopy, polarimetry, etc.).

To search for the most stable conformations of biscamphorolidenpropylenediamine (CPDA), we scanned the energy profile during rotation of dihedral angles D1, D2, D3, D4. For the found “stable conformations” additional geometry optimization was carried out using density functional theory, CAM-RB3LYP functional basis set of 631G(d,p) included geometry optimizations followed by computations of harmonic vibrations. Force-field calculations yielded no imaginary frequencies, indicating that the optimized configurations correspond to the minima on the potential energy hypersurfaces.

**Figure 1:**
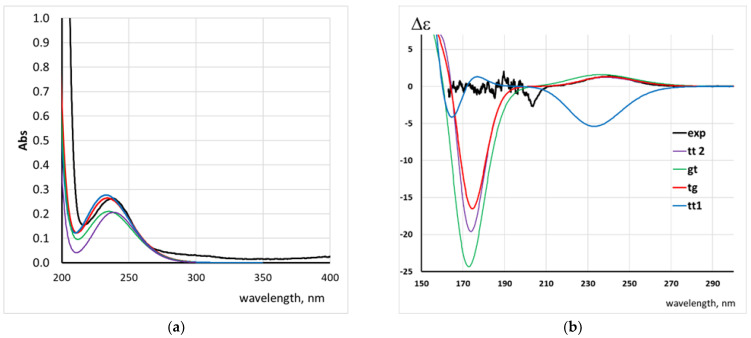
UV/Vis (**a**) and ECD (**b**) spectra of CPDA in ethanol at 25 °C. Experimental (black) and calculated conformers (color).

As a result of quantum chemical simulation, the structures of the most energetically stable conformers were calculated; their energies and internal rotation angles are listed in Table 1. The electronic circular dichroism (ECD) spectra of flexible chiral molecules depend on their stereochemistry in terms of both the absolute configuration and the molecular conformation, which very sensitively determines the overall shape of the spectrum, including the sign. The uniquely high sensitivity of ECD to conformation makes this technique a useful alternative or addition to standard conformational spectroscopic instruments such as NMR [30]. In this regard, on the basis of quantum-chemically optimized structures, the ECD and UV/Vis spectra of the four most stable conformers were calculated (Table 1) (see experimental section) and presented in Figure 1 together with the experimental CPDA spectra.

Figure 1 reveals a low sensitivity of UV/Vis spectra to conformational changes in camphorodiimine, which is confirmed by TD-FT calculations (Table S3), but significant differences are observed in the ECD spectrum, indicating a change in the sign of CD in the trans-trans conformer (tt-1) compared to other stable structures (Table 1). This allows to exclude tt-1 from further consideration. As for the rest of the conformers, the signs of the CD bands for them coincide with those obtained experimentally (Figure 1). The discrepancy in the short wavelength region between the experimental and calculated CD spectra in Figure 1b may be related to the difficulty in obtaining precise spectra in the vacuum ultraviolet range of shorter than 220 nm due to the light source, the absorption by solvent, and the sample cell spectra. For the conformers tt-2, tg and gt it is necessary to use other relevance criteria. Another fundamental characteristic of optically active substances is the specific angle of optical rotation [α], the experimental value of which for CPDA, as well as the calculated values for conformers are given in Table 1. These data indicate that the [α] values for the gt and tt-2 conformers, although they coincide in sign with the experimental value (−29.6°), are much higher than the later. The highest agreement is observed for the tg (−30.7°) conformer, which also has the lowest energy.

Taking into account the presence of local dipoles of molecular moieties in the CPDA, primarily, the C=N imine bond, it was of interest to calculate the dipole moments of the conformers and compare them with the experimental value.

**Figure 2:**
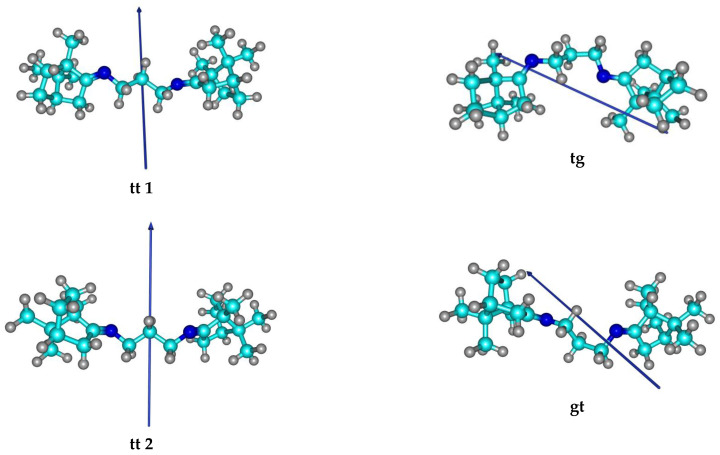
Structure of CPDA conformers and direction of dipole moments.

These data (Table 1 and Figure 2) indicate that the presence of one gauche conformation in the aliphatic linker leads to a change in the direction of the resulting dipole moment from mutually perpendicular with respect to the long molecular axis to predominantly mutually parallel (Figure 2). At the same time, the value of the dipole moment of the tg conformer 2.29 D is closest to the experimental value 3.15 D (Table 1).

One of the most informative tools for studying the structure of conformers is NMR [25]. Therefore, ^1^H, ^13^C, and ^15^N NMR spectra were obtained and presented in Supplementary Materials (Figures S1–S4) and the corresponding theoretical spectra were calculated on the base of the optimized conformer structures (Figure 2) by the GIAO method. Experimental and calculated chemical shifts are listed in Tables S1 and S2 in Supplementary Materials, on the basis of which the average deviations between them were calculated for all nuclei ^1^H, ^13^C and ^15^N, presented in Table 2.

The data in Table 2 show that the smallest deviation of the calculated chemical shifts from the experimental ones is revealed for the tg conformer. In addition, two resonance signals in the experimental ^15^N NMR spectrum (Table 2) indicate the chemical nonequivalence of nitrogen nuclei, i.e., the presence of the C-C bond g-conformation in the structure of the predominant conformer in solution. Thus, the results of quantum-chemical calculations, ECD, and NMR spectroscopy, polarography, and measurements of dipole moments convincingly indicate the predominant contribution of the tg conformer in the gas phase and in solutions of biscamphorolidenpropylenediamine.

In addition to conformational effects, the chirality of optically active compounds can also be strongly influenced by their supramolecular state [31]. Associative processes in such systems can result in supramolecular helical assemblies formed from chiral and achiral small molecules, oligomers (foldamers) and helical and nonhelical polymers from the viewpoints of their formations with unique chiral phenomena, such as amplification of chirality during the dynamic helically assembled processes, properties, and specific functionalities [32].

**Figure 3:**
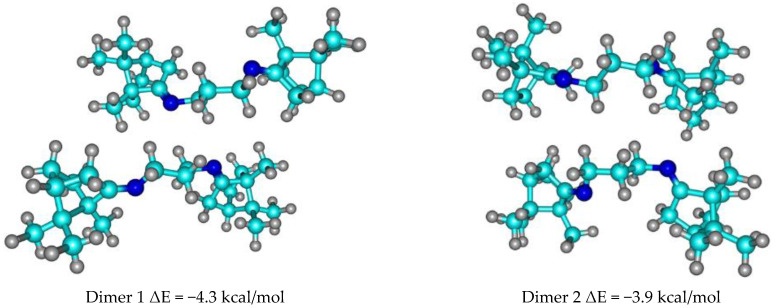
Structure of CPDA dimers in the tg conformational state.

The transfer of chirality during associative processes creates opportunities for the phenomenon of chiral memory and chiral separation [33]. Taking this into account, quantum-chemical simulation of the supramolecular dimerization of bis-camphorolidenpropylenediamine (CPDA) in its most stable tg-conformation was carried out (Figure 2). As a result, two most stable structures were obtained (dimer 1 and dimer 2, Figure 3), the calculated characteristics of which are given in Table 1, the ECD spectra in Supplementary Materials Figure S5).

Both dimers have similar energies of formation from monomeric molecules and calculated ECD spectra, which differ little from the spectrum of the tg conformer (Supplementary Materials Figure S5). In this case, the values of the specific optical rotation and dipole moment of the associates are the criteria for the dimerization probability. These data, presented in Table 1, indicate a significant decrease in the dipole moment of dimer 1 due to compensation of the dipoles of the tg conformer. In this case, the value μ_dim2_ = 3.10 D practically coincides with the experimental value (3.15 D). The values of the optical rotation of dimer 2 (29.6°) and the experimental value (−29.6°) are also close, in contrast to [α]_dim1_ = −47.9° (Table 1). These data suggest that a significant contribution of type 1 dimers to the association of camphor-containing diimine CPDA is unlikely.

**Figure 4:**
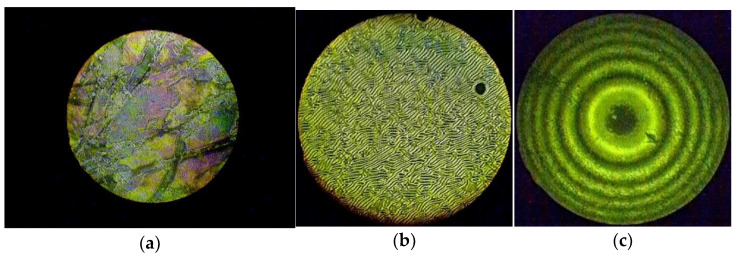
Marble texture of CB-2 at 70.1 °C (**a**). Fingerprint texture of CB-2 + 8.7% CPDA mixture at 45 °C (**b**) and Newton’s rings system of CB-2 + 5.263% CPDA mixture at 35.8 °C (**c**).

The behavior of conformationally flexible chiral dopants during helix induction in nematic liquid crystals is of particular interest. In this work, the mesomorphic and physical properties of a liquid crystal based on a binary eutectic mixture of alkoxycyanobiphenyls (CB-2) doped with camphorodiimine CPDA were studied (see the experimental section). The introduction of this dopant in an amount of several percent into a nematic LC leads to twisting the helix, as evidenced by the fingerprint texture typical of a chiral nematic in polarized light [34] and the interference pattern with Newton’s rings (Figure 4).

**Figure 5:**
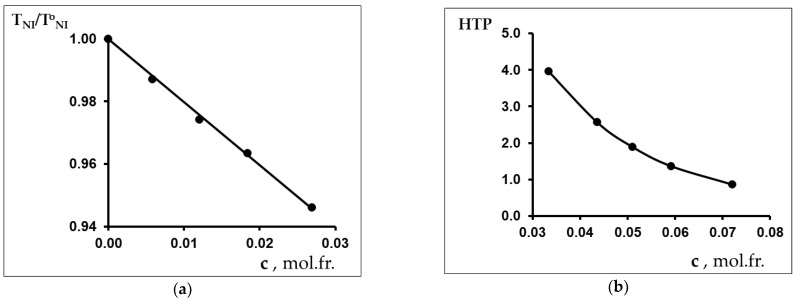
Fragment of the phase diagram of the CB-2+CPDA system (**a**) and dependence of HTP on the dopant concentration at 33 °C (**b**).

Polarization microscopy was used to measure the clearance temperatures of a chiral nematic (phase transition N*→I) with different contents of a chiral dopant, presented as a fragment of the phase diagram (T_NI_/T°_NI_ = f(c), where T°_NI_ is the clearance temperature of the initial LC, c is the molar fraction of dopant (Figure 5a). These results were confirmed by DSC data (Figure S6).

The slope of the straight line β = d(T_NI_/T°_NI_)/dc (Figure 4a) was calculated, which is the degree of dopant effect on the thermal stability of the mesophase (Table 3). On the base of the quantitative analysis of the system of Newton’s rings by the Grandjean-Cano method (see the experimental section), the values of the helix pitch of chiral nematics were measured and the values of the helical twisting power (HTP) were calculated using the equation:

HTP = (p · c · r)^−1^ (1)

where p is the pitch of the helix, c is the mole fraction of the dopant, and r is its enantiomeric purity.

The concentration dependences of HTP are shown in Figure 5b, and the maximum HTP values are listed in the table. 3. It should be noted that the helix pitch determined from the interference pattern (Figure 4c) coincides with the values measured directly from the fingerprint texture within the experimental error. The decrease in the HTP of the nematic phase with increasing dopant concentration (Figure 5b) is apparently connected with the CPDA association process, as shown in Figure 2 and Table 1, and with a decrease in the efficiency of chirality transfer from the associate to the LC compared to the monomer CPDA.

The camphor moiety (1,7,7-trimethylbicyclo [2.2.1]heptan-2-ylidene group) in azo derivatives is responsible for their antiviral activity [17,18]. This chiral moiety containing stereogenic centers also ensures the transfer of chirality to the matrix of nematic LCs. In this regard, it is of undoubted interest to evaluate the effect of azo-containing synthons on the effect of chiral camphor-containing dopants with respect to nematic LCs based on mixtures of cyanobiphenyls. For this, Table 3 shows the values of the specific optical rotation of camphor-containing chiral dopants, the degree of their effect on the thermal stability and the efficiency of twisting of the nematic mixtures of cyanobiphenyls. Dinitrile CDPC was shown to have the least destabilizing effect on the mesophase, apparently due to its high polarity (6.8 D [26]) and efficient incorporation into the polar nematic matrix of cyanobiphenyls. The symmetrical bulky low-polar dicamphor-containing hemiporphyrazine HPA destabilizes LC to the greatest extent, but has a four times higher HTP value compared to its precursor, due, among other things, to higher optical activity [28]. The stereochemically non-rigid dicamphor-containing diimine CPDA, having a low optical activity and moderately destabilizing the LC, nevertheless has a higher HTP value compared to its analogues CDPC and HPA, which may be due to its conformational compliance adaptive to the orientationally ordered liquid–crystalline matrix.

The use of helical LCs in various fields arouses interest in the anisotropic physical properties of chiral nematic phases induced by chiral dopants. In this regard we studied the dielectric and optical properties of CPDA solutions in CB-2.

**Figure 6:**
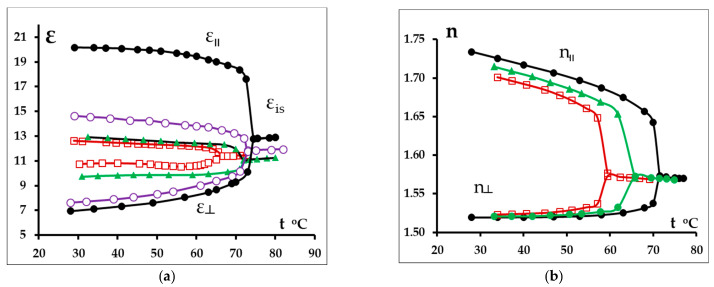
The effect of temperature and dopant concentration on the components of dielectric permittivity (**a**) and birefringence (**b**) of CPDA solutions in CB-2. (●—CB-2; ○—0.712%; ▲—1.107%; **□**—3.29%).

The temperature dependences of the dielectric constant components (ε_║_, ε_┴_, ε_is_ ) and refractive indices (n_║_, n_┴_, n_is_) of CPDA solutions in the nematic LC (Figure 6) indicate a strong influence of the chiral dopant on the physical properties of both the chiral nematic and its isotropic phase. Doping with even small additions of CPDA has the strongest effect on the dielectric constant of the LC (Figure 5a), sharply lowering the anisotropy ∆ε = ε_║_ − ε_┴_ due to 3D compensation of the polar LC dipoles in the helical structure [26]. Modification with a chiral dopant also reduces the birefringence ∆n = n_║_ − n_┴_ mainly due to a decrease in the parallel component n_║_ owing to a decrease in the orientational order parameter.

## 3. Materials and Methods

Bis(camphoroliden)propylenediamine (CPDA) was used as a chiral dopant, which was synthesized according to the following procedure [24]: 4.74 g (0.064 mol) of 1.3-propylenediamine and 0.4 g of p-toluenesulfonic acid (6.56% mol on camphor) were added to a solution of 10 g (0.032 mol) (+) camphor in toluene (150 mL). The reaction was refluxed for 5-12 h until the azeotrope was completely distilled using a Dean-Stark trap. After completion of the reaction, the mixture was washed with acidified water, then with water and dried over Na_2_SO_4_ and the solvent was removed using a rotary evaporator. The resulting product was purified by column chromatography on 40 g SiO_2_, eluent hexane and ethyl acetate. Product yield 73%. The resulting compound was characterized using elemental analysis, NMR and UV spectroscopy: ^1^H NMR (δ, ppm, №, J/Hz): 3.23 m (4H, 12,13); 2.28 ddd (2H, 2″,16″, J_2_ = 16.9, J_3_ = 3.8), 1.88 t (2H, 4,18, J_3_ = 4.5Hz); 1.81 m (6H, 2′,16′, 6″,20″, 25) 1.61 ddd (2H, 5″,19″, J_2_ = 13.0, J_3_ = 4.2 Hz); 1.30 ddd (2H, 5′,20′, J_2_ = 13.5, J_3_ = 4.3 Hz); 1.15 ddd (2H, 6′,20′, J_2_ = 12.5, J_3_ = 4.2 Hz), 0.92 s (H6,10,24), 0.88 s (H6,8,22), 0.70 s (H6,9.23). ^13^C NMR (δ, ppm, №): 181.64 (1,15); 53.40 (3,17); 50.15 (12,13); 46.84 (7,21); 43.80 (4,18); 35.33 (2,16); 32.18 (5,19); 31.44 (25); 27.48 (6,20); 19.56 (8,22); 18.98 (9,23); 11.47 (11,47). λmax: 238.6 nm Anal. Calcd. for C_23_N_2_H_38_ (342.30): C, 80,63%; H, 11.19%; N, 8.18%. Found: C, 80.4%; H, 10.8%; N, 8.4%. MS (MALDI-TOF, dithranol): m/z 343.7 [M + H]+.

Liquid–crystalline mixtures based on cyanobiphenyl derivatives (CB-2) were used to study chiral induction. The nematic mixture CB-2 is composed of two components: 55% 4-pentyloxy-4′-cyanobiphenyl (5OCB) and 45% 4-heptyloxy-4′-cyanobiphenyl (7OCB) (Qualification “Pure for analysis” Reahim) The phase transition temperatures of CB-2 are as follows: crystal → 22 °C → nematic → 74.5 °C → isotropic [35]. Liquid–crystalline mixtures with dopants were prepared gravimetrically by mixing at temperatures corresponding to the isotropic liquid state. At all the temperatures of the study, separation of mixtures was not observed, which was recorded by the polarization microscopy.

### 3.1. Measurement of UV/Vis, CD Spectra

Circular dichroism (CD) spectra of CPDA solutions were recorded on a «Jasco 1500» spectrometer (JASCO Corporation Ishikawamachi Hachioji-shi, Tokyo Japan). UV/Vis spectra were obtained on a Perkin Elmer «Lambda 20» scanning spectrophotometer (Markham, ON, Canada).

### 3.2. NMR Measurement

^1^H, ^13^C NMR, HMBC ^15^N-^1^H spectra were recorded on “Avance III Bruker 500” NMR spectrometer with operating frequencies of 500.17, 125.77 MHz and 50.68 MHz, respectively. A 5 mm 1H/31P/D-BBz-GRD Triple Resonance Broad Band Probe (TBI) was employed. To assign the NMR signals in the spectra, two-dimensional methods COSY, HSQC and HMBC were used.

The two-dimensional ^15^N-^1^H HMBC spectra were recorded with 1024 t1 increments using the short-range ^1^H-^15^N HMBC pulse sequences instead of direct HSQC because Bruker pulse programs has some failures with registration of ^1^H-^15^N HSQC. On the other hand, the one bond correlations between ^15^N and directly attached ^1^H are not filtered out in HMBC and we might also observe HSQC correlation too. Nitromethane signal was used as the ^15^N chemical shift reference; the sweep width was 601 ppm in the nitrogen dimension and 15 ppm in the proton dimension. To diminish the experimental duration, the HMBC spectra were recorded using non-uniform sampling (NUS value 25%) [36].

### 3.3. Optical Rotation and Dipole Moment

The specific optical rotation of CPDA in ethanol was measured on a «Polartronik V202» polarimeter (Schmidt-Haensch, Germany) at 589.44 nm.

The dipole moment of the dopant was determined by the Guggenheim and Smith method using data on the permittivity and refractive index of solutions in carbon tetrachloride [37].

### 3.4. Quantum-Chemical Calculations

The structures of CPDA conformers and dimers were optimized by using the CAM-B3LYP functional of the Gaussian 09W [38] software package with base sets of 6-31G (d, p). (The optimized Cartesian coordinates are available in the Supplementary Materials). The search for stable CPDA conformers was performed by varying the torsion angles D2,D3 using the semi-empirical AM1 method, followed by optimization at stationary points using the DFT/CAM-B3LYP/6-31G(d,p) and computation of harmonic vibrations method. Analytic Hessian calculations indicated the absence of the imaginary vibrational frequencies and, therefore, the optimized structures corresponded to the minima on the PES.

TD-DFT calculations were then carried out using the CAM-B3LYP/6-31G(d,p) scrf = (cpcm, solvent = ethanol) functional since it contains a long-range correction. The number of excited states was 20. NMR shielding constants were calculated using the GIAO method [39] with basis CAM-B3LYP/6-311++(d,p) scrf = (cpcm, solvent = chloroform). Chemcraft software [40] was applied for the preparation of input data files, as well as for processing and visualization of the computed results.

### 3.5. Measurement of Phase Transitions Temperatures

The study of the mesomorphic properties was carried out by polarization microscopy on a «Polam P211» polarizing microscope equipped with a thermotable. The phase transition temperatures were measured with an accuracy of ±0.1 °C. To obtain textures, a cell of two plane-parallel glasses was used without special surface treatment.

### 3.6. Measurement of Pitch

The helical pitch of the chiral phases at various temperatures and dopant concentrations was measured by the Grandjean–Cano method [41] using a convex lens and a plane-parallel plate at a monochromatic light wavelength of 551 nm. For the CB-2 + CPDA system at some temperatures and dopant concentration, the helix pitch was directly measured from the “fingerprint” textures obtained with a camera-mounted microscope [42]. The pitch values measured by the two methods coincided within the limits of the experimental error. The ability to form a spiral mesophase was described by the helical twisting power (HTP) according to Equation (1). The error in determining HTP was ±1 μm^−1^.

### 3.7. Measurement of Permittivity

The permittivity components were measured similarly to [26,28] using an LCR-817 (INSTEK) at a frequency of 10 kHz, with a cell voltage of 1 V, parallel (ε_║_) and perpendicular (ε_┴_) to the direction of the 1600 Gauss magnetic field. The error in determining ε did not exceed ±0.02.

### 3.8. Measurement of Refraction Indices

The refraction indices (n_0_ = n_┴_) for the mesomorphic state and isotropic-liquid phase (n_is_) were measured with an Abbe temperature-controlled refractometer at the wave length of 589 nm with an accuracy of ±0.0005. The prism surfaces of the refractometer were rubbed for sample orientation. The refraction index ne = n_ǁ_ was calculated using the relation for the average value: n^2^ = 1/3 × (n_e_^2^ + 2 × n_o_^2^) which was determined by extrapolation of n_is_ to the region of nematic phase taking into account the temperature dependence of the LC density. The birefringence of the liquid–crystal material was determined as Δn = n_e_ − n_0_ = n_ǁ_ − n_┴_. The error in determining Δn did not exceed ±0.001.

## 4. Conclusions

The article presents the results of an experimental and theoretical study of the conformational and associative state of biologically active bis-camphorolidenpropylenediamine (CPDA). Based on ECD, ^1^H, ^13^C, ^15^N NMR spectroscopy data, polarimetry and dipole moment measurements, as well as quantum chemical calculations, the most probable conformational state (tg) of CPDA was established. Quantum-chemical simulation of the association allowed for the conclusion that the structure of the CPDA dimer is the most stable with a predominantly mutually parallel arrangement of molecular dipoles, which was confirmed experimentally. Polarization microscopy revealed the formation of “fingerprint” textures and a system of Newton’s rings when doping a nematic LC based on a mixture of cyanobiphenyls with bis-camphorolidenpropylenediamine, which indicates the induction of a helical mesophase. Clearance temperatures (T_N*I_) were measured at various concentrations of the dopant, and the degree of its influence on the thermal stability of the mesophase (β) was determined. The system of rings was used to measure the helix pitch by the Grandjean–Cano method and to calculate the helical twisting power (HTP). The influence of the dopant association on the decrease in HTP was found. The effect of camphor-containing chiral dopants of various structures, such as conformationally flexible bis-camphorolidenpropylenediamine, polar camphor-substituted 2,3-dicyanopyrazine, and symmetrical dicamphor-substituted hemiporphyrazine, on nematic LCs was analyzed.

The values of the permittivity and birefringence components of CPDA solutions in CB-2 were experimentally measured. A strong influence of the dopant on the anisotropic physical properties of the induced chiral nematic was established. A sharp decrease in the permittivity anisotropy was shown upon the introduction of small amounts of CPDA due to the formation of a helix and compensation of the molecular dipoles of the polar LC.

## Figures and Tables

**Table 1:** Results of quantum-chemical calculations of CPDA conformers and dimers.

Conformers	E au	μ, D *	[α] **	D1	D2	D3	D4
tt-1	−1008.2841	1.76	−14.1	132.0	176.0	176.0	132.2
gt	−1008.2849	1.21	−132.9	−125.0	−59.5	177.2	144.9
tt-2	−1007.9500	1.33	−172.7	−119.5	−174.4	−174.4	−119.5
tg	−1008.2850	2.29	−30.7	126.5	178.6	64.2	175.8
dimer 1	−2016.5770	0.61	−47.9				
dimer 2	−2016.5763	3.10	−27.9				

* Experimental value of dipole moment 3.15 D. ** (5893.0 A) Experimental optical rotation angle −29.6°.

**Table 2:** ^15^N NMR spectrum of CPDA in CDCl_3_ and average total deviations of the calculated values of the ^1^H and ^13^C chemical shifts of the conformers from the experimental ones.

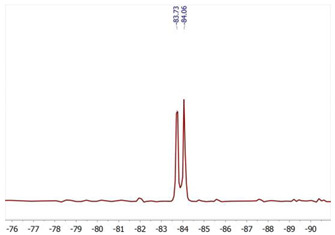				
**Nucleus**	**Average Deviation, ppm for ** **Conformer**			
	**tt-1**	**gt**	**tt-2**	**tg**
^1^H	0.087	0.139	0.177	0.057
^13^C	4.90	4.16	5.08	4.11

**Table 3:** The specific optical rotation of camphor-containing chiral dopants and the degree of influence on the thermal stability and twisting efficiency of cyanobiphenyl nematic mixtures.

Chiral Dopant	[α], ^o^ (5893.0 A)	*β*, (mol.fr.)^−1^	HTP, (μm)^−1^
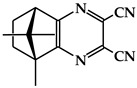 **CDCP**	+22.4 *	−1.16 *	0.57 *
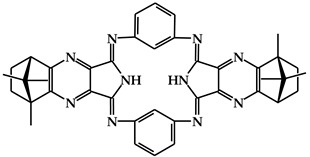 **HPA**	+88.0 **	−6.24 **	2.19 **
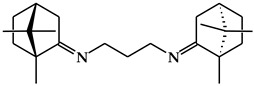 **CPDA**	−29.6	−2.02	3.16

* [26]; ** [28].

## Data Availability

Experimental and calculated data on the basis of which the results of this article were discussed are given in the Supplementary Materials.

## Supplementary Materials

The following supporting information can be downloaded at: https://www.mdpi.com/article/10.3390/molecules28052388/s1, Figures S1–S4: NMR spectra; Table S1: Correlation between experimental and calculated (GIAO/cam-b3lyp/6-311++g(d,p) scrf = (cpcm, solvent = chloroform)) chemical shifts of 1H (bis)camphoralidene-propylenediamine; Table S2: Correlation between experimental and calculated (GIAO/cam-b3lyp/6-311++g(d,p) scrf = (cpcm, solvent = chloroform)) chemical shifts of 13C (bis)camphoralidene-propylenediamine; Figure S5: Calculated (TD-DFT CAM-B3LYP/6-31G(d,p) scrf = (cpcm, solvent = ethanol N = 20) spectra ECD of dimer CPDA; Figure S6: DSC thermograms of CB-2 and CD-2+CPDA mixtures; Figure S7: MALDI-TOF MS of CPDA; Table S3: Results of TD-DFT calculations Cartesian coordinates of CPDA conformers.
