# Supplementary material for: Conformational and Supramolecular Aspects in Chirality of Flexible Camphor-Containing Schiff Base as an Inducer of Helical Liquid Crystals
Source: Molecules. 2023 Mar 5;28(5):2388. doi: 10.3390/molecules28052388 (PMC10005677; doi:10.3390/molecules28052388)
Supplement: Supplementary file 1 [file molecules-28-02388-s001.zip › molecules-2206648-supplementary.pdf]

Supplementary Materials

for

Conformational and Supramolecular Aspects in Chirality of Flexible Camphor-Containing Schiff Base as an Inducer of Helical Liquid Crystals

Vladimir Burmistrov *, Alena Batrakova, Viktor Aleksandriiskii *, Igor Novikov, Konstantin Belov, Ilya Khodov, Oskar. Koifman

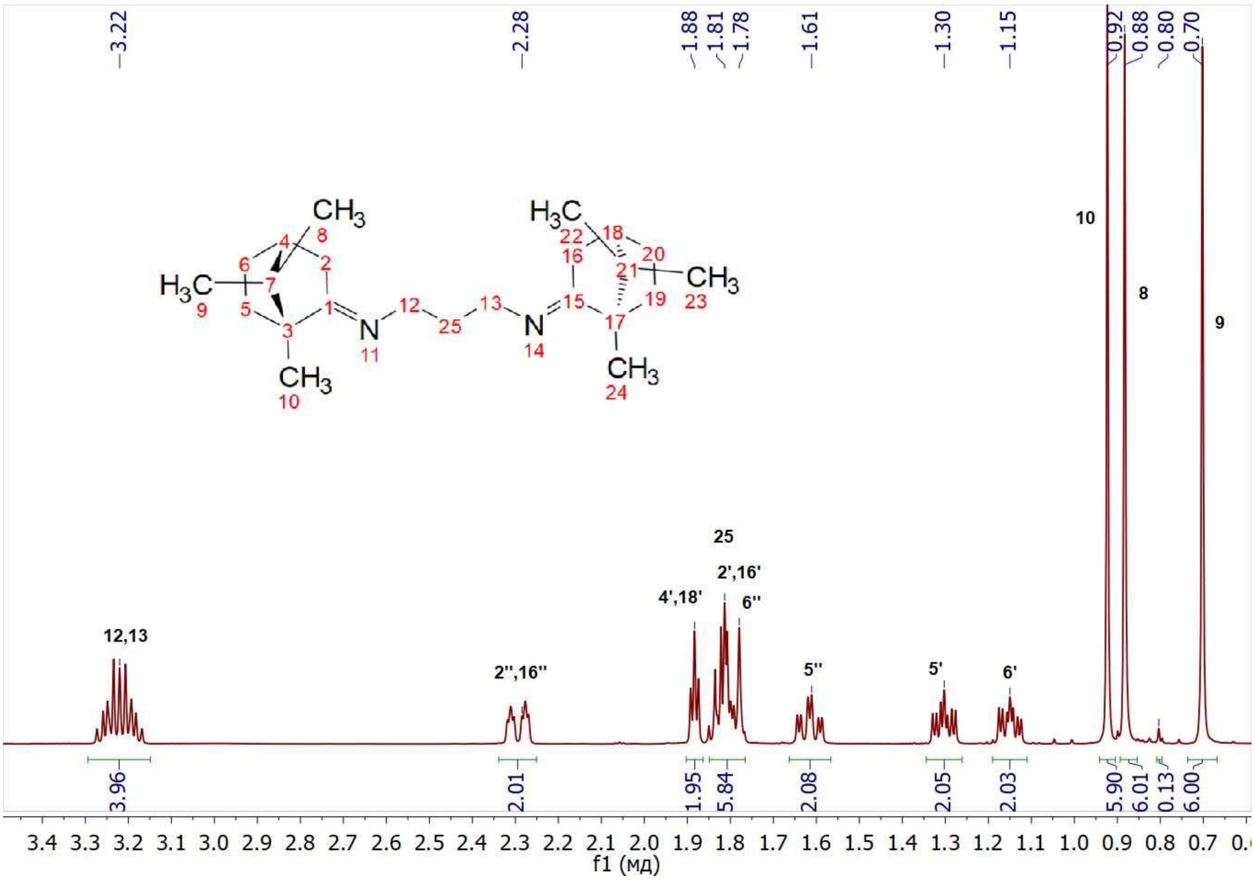

Figure S1. ^{1}H NMR spectrum of CPDA in $CDCl_3$

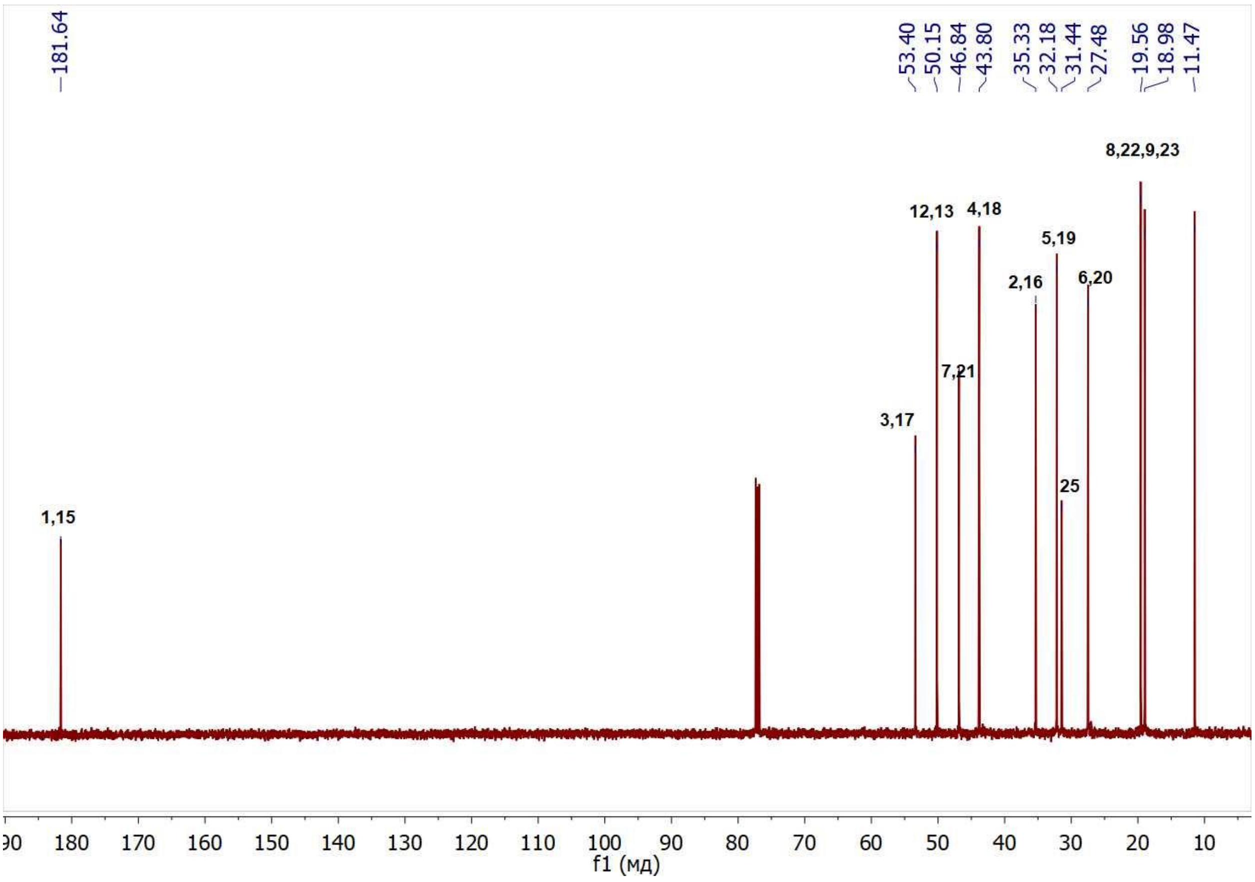

Figure S2 ^{13}C NMR spectrum of CPDA in $CDCl_3$

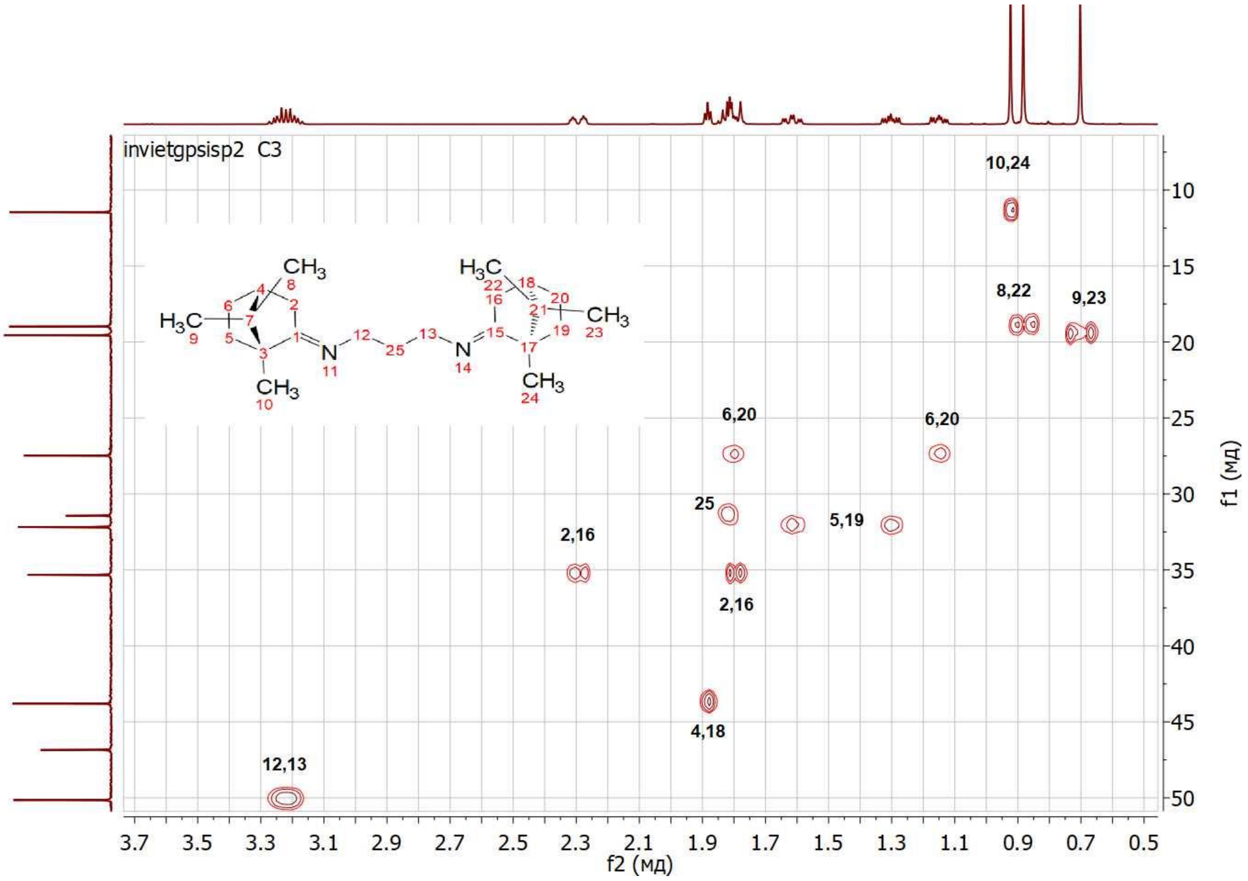

Figure S3. HSQC ^{13}C-^{1}H NMR spectrum of CPDA in $CDCl_3$

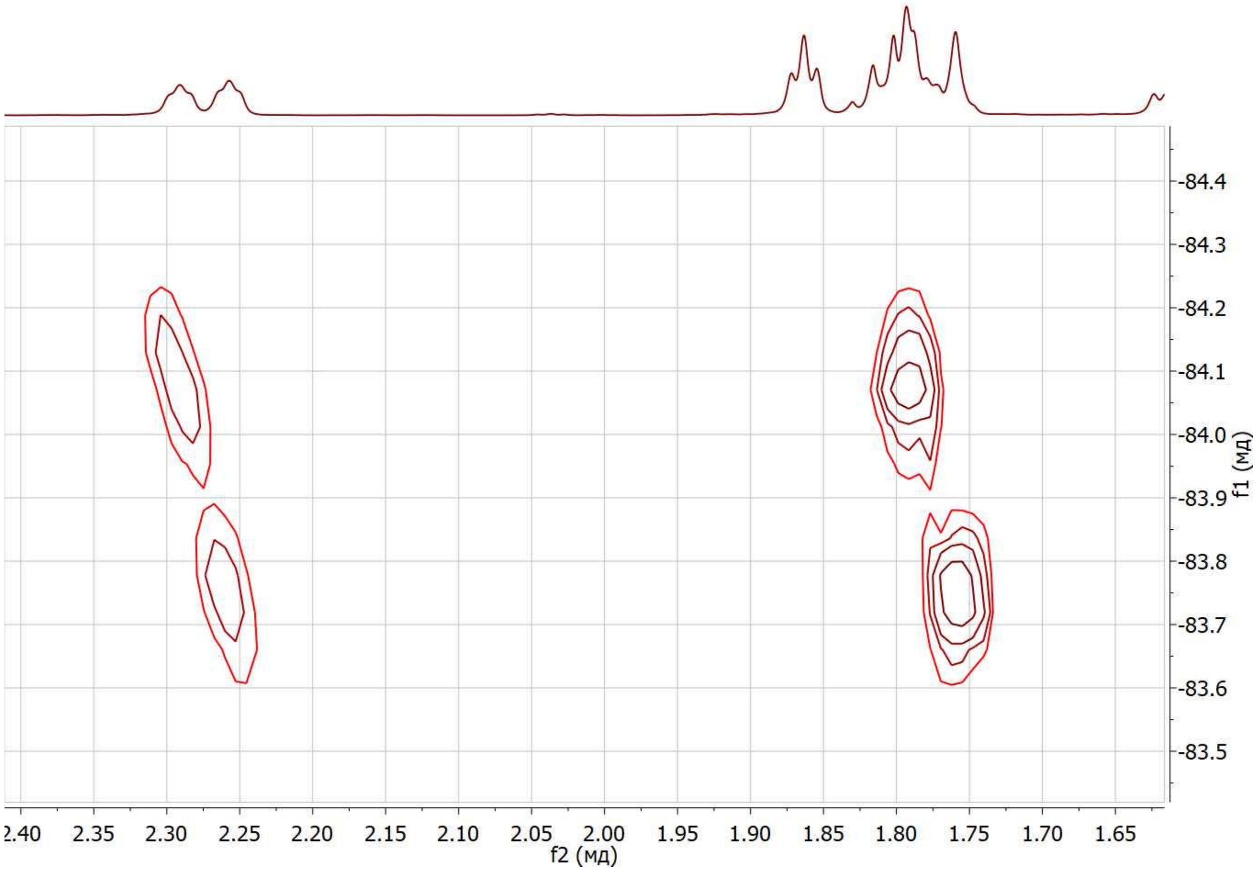

Figure S4. HMBC ^{15}N-^{1}H NMR spectrum of CPDA in $CDCl_3$

Table S1 Correlation between experimental and calculated (GIAO/cam-b3lyp/6-311++g(d,p) scrf=(cpcm,solvent=chloroform)) ^{1}H chemical shifts of (bis)camphoralidene-propylenediamine

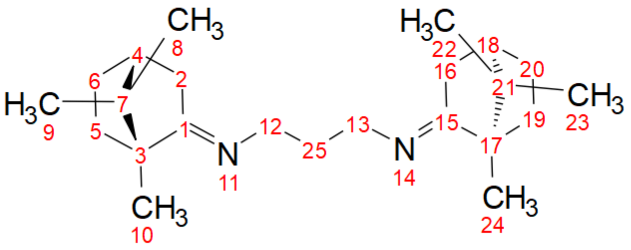

	exp	GIAO			
№ ^{1}H	CDCl3	**tg**	**tt 2**	**gt**	**tt 1**
12"	3.23	3.283	3.612	3.636	3.416
13"	3.23	3.352	3.612	4.004	3.415
12'	3.21	3.200	3.532	3.379	3.216
13'	3.21	3.063	3.532	3.412	3.213
2"	2.28	2.333	2.495	2.376	2.483
16"	2.28	2.303	2.495	2.547	2.484
4	1.88	1.878	1.996	1.760	1.903
18	1.88	1.970	1.996	1.925	1.901
25	1.81	1.950	1.812	1.807	1.846
2'	1.79	1.904	1.916	2.127	1.867
16'	1.79	1.705	1.916	1.752	1.869
6"	1.8	1.826	1.838	1.935	1.916
20"	1.8	1.854	1.838	1.922	1.917
5"	1.61	1.711	1.909	1.733	1.697
19"	1.61	1.781	1.909	1.722	1.697
5'	1.3	1.335	1.461	1.304	1.266
19'	1.3	1.261	1.461	1.305	1.266
6'	1.15	1.204	1.293	1.291	1.254
20'	1.15	1.212	1.293	1.204	1.257
10	0.92	1.016	1.060	0.965	0.978
24	0.92	1.041	1.060	0.861	0.978
8	0.88	0.960	1.060	0.868	0.813
22	0.88	0.944	0.865	0.861	0.812
9	0.7	0.868	0.935	1.114	1.020
MAE		0.057	0.177	0.139	0.087
R^2		0.992	0.989	0.964	0.983

Table S2 Correlation between experimental and calculated (GIAO/cam-b3lyp/6-311++g(d,p) scrf=(cpcm,solvent=chloroform)) ^{13}C chemical shifts of (bis)camphoralidene-propylenediamine

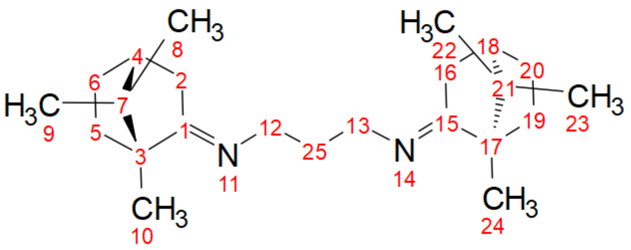

	exp	GIAO			
№ ^{13}C	CDCl3	**tg**	**tt 2**	**gt**	**tt 1**
1	181.64	184.234	187.072	184.755	184.529
15	181.64	188.413	187.072	184.598	184.548
3	53.404	59.020	60.565	59.354	59.749
17	53.404	59.239	60.565	59.239	59.759
12	50.151	57.162	57.160	54.757	56.249
13	50.151	53.223	57.161	56.288	56.241
7	46.844	52.055	53.303	52.363	51.869
21	46.844	52.516	53.303	51.865	51.870
4	43.802	48.414	51.041	46.704	48.826
18	43.802	49.803	51.041	49.853	48.782
2	35.333	39.927	39.223	39.950	39.926
16	35.333	39.753	39.222	39.222	39.981
5	32.180	36.794	36.891	36.953	36.099
19	32.180	35.351	36.892	36.357	36.099
25	31.435	39.466	38.839	39.741	40.647
6	27.484	31.398	31.489	30.733	36.099
20	27.484	32.158	31.489	30.687	36.099
8	19.564	21.680	22.662	21.791	21.914
22	19.564	22.115	22.661	21.705	21.906
9	18.979	21.527	21.560	21.369	21.705
23	18.979	21.280	21.560	21.128	21.711
10	11.469	14.531	14.553	14.189	14.616
MAE		4.1082	5.0757	4.1617	4.9007
R^2		0.9987	0.9986	0.9987	0.9979

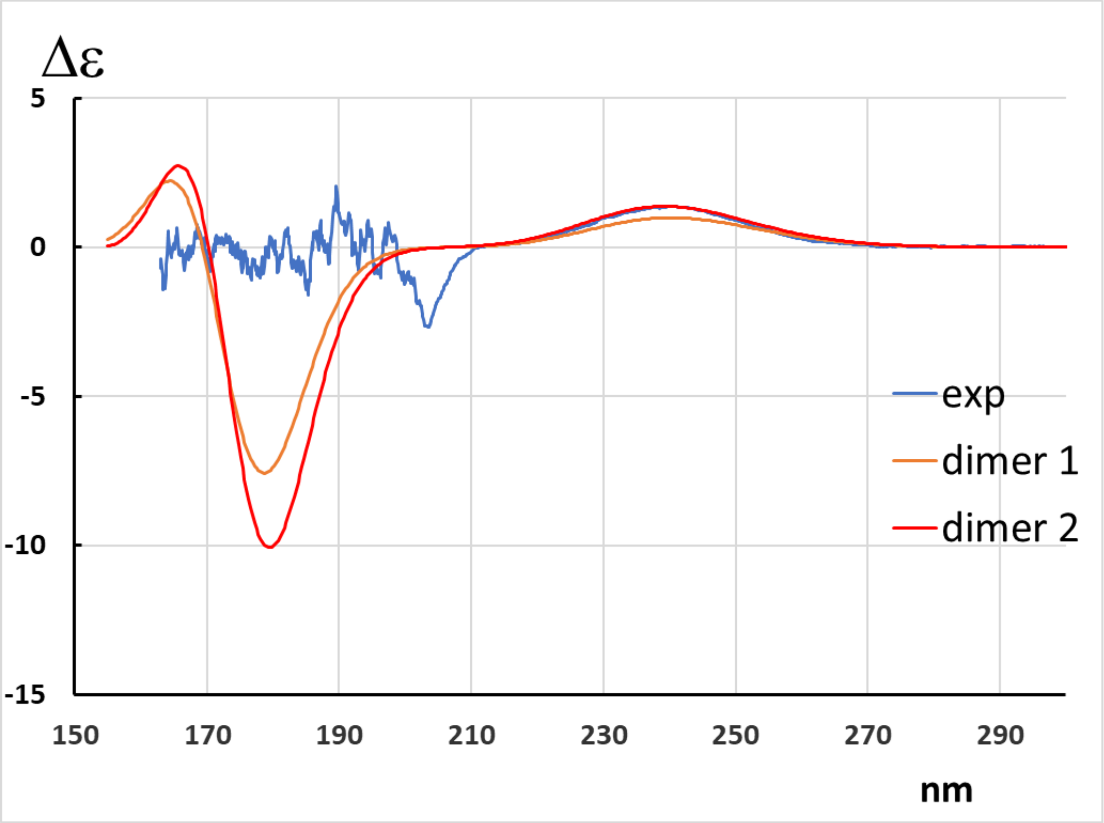

Figure S5. Calculated (TD-DFT CAM-B3LYP/6-31G(d,p) scrf=(cpcm, solvent=ethanol N=20) spectra ECD of CPDA dimers

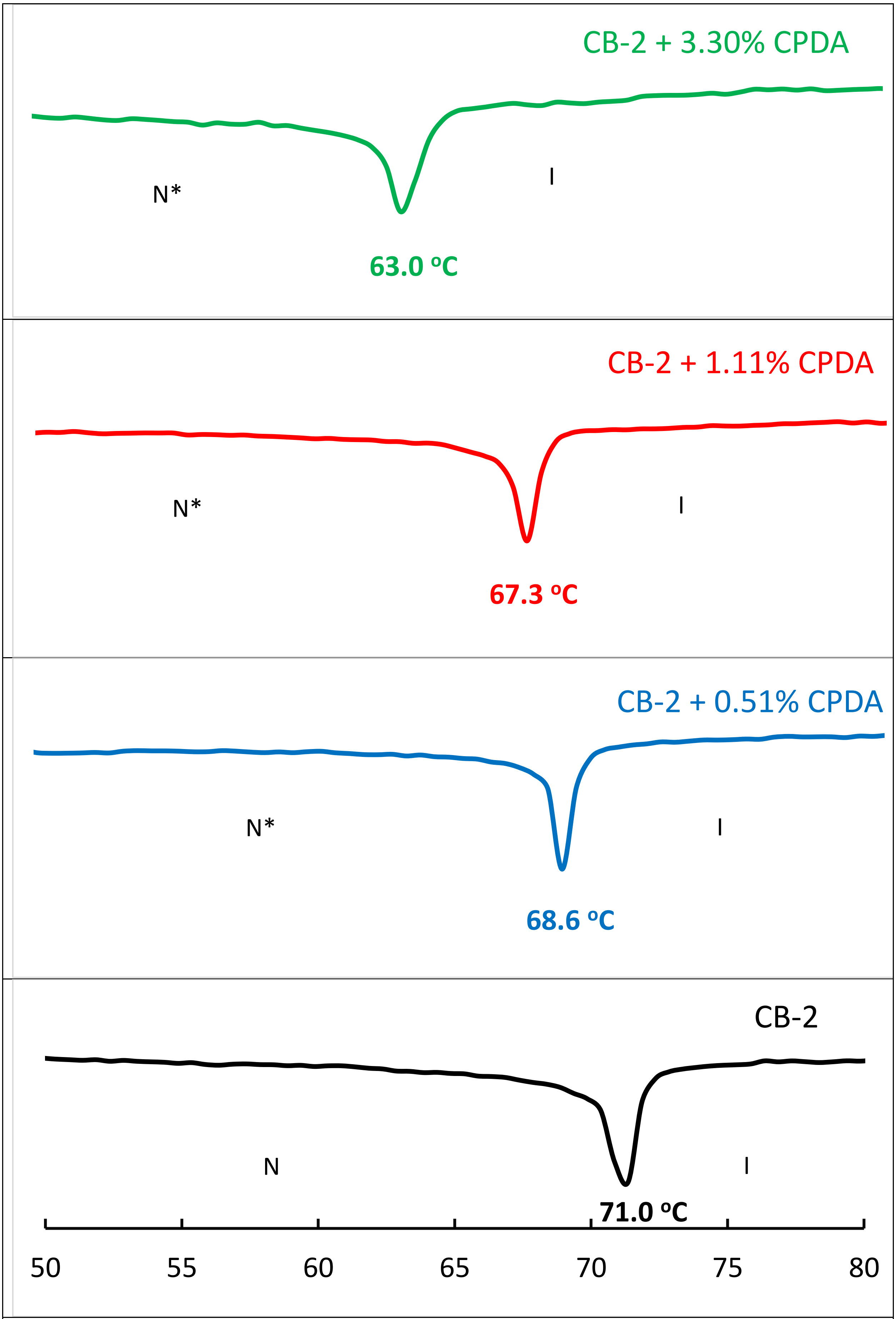

Figure S6. DSC thermograms of CB-2 and CD-2+CPDA mixtures upon heating with rate 1°C/min. (NETZSCH STA 449F3)

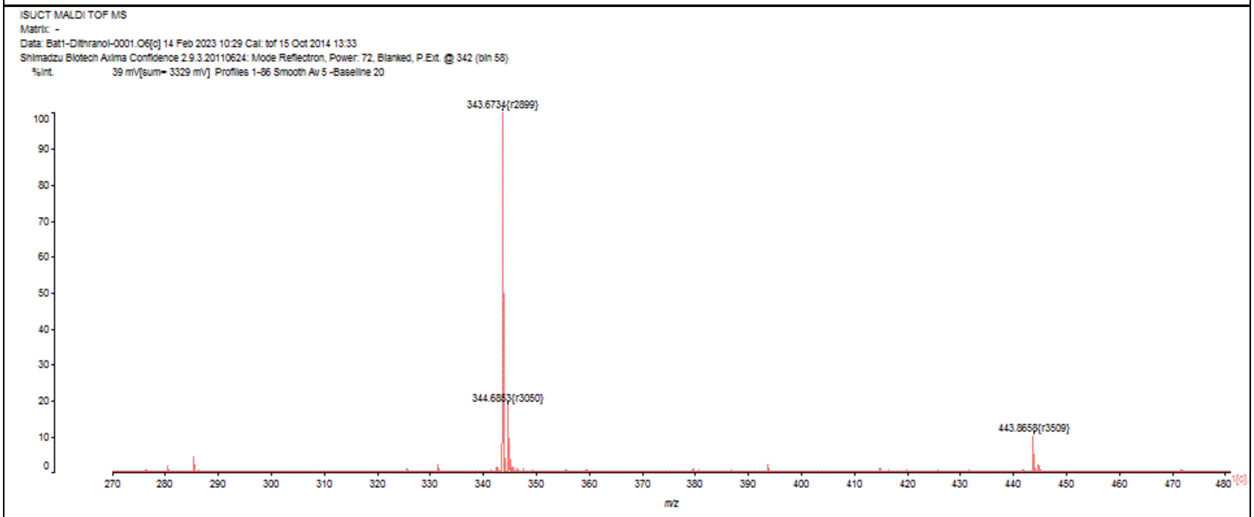

Figure S7. MALDI-TOF MS of CPDA (Dithranol, Jupiter Shimadzu Axima Confidence).

Table S3. Results of TD-DFT calculations of the electronic transitions along with their energy, oscillator strength (f), and major orbital contribution.

	λ exp, nm	λ calc, nm	f	excitation energies, eV	Assignment[1]
tt1	-	168.76	1.1443	7.3467	H-3→L+1; H-2→L+1 (π–π*)
	239.2	232.88	0.0023	5.3239	H-1→L; H→L+1 (n–π*)
gt	-	170.38	1.065	7.2767	H-3→L+1; H-2→L; H→L+1 (π–π*)
	239.2	233.51	0.0024	5.3096	H-1→L; H-1→L+1; H→L+1 (n–π*)
tt2	-	177.93	0.9264	6.9683	H-3→L+1; H-2→ ; H→L (π–π*)
	239.2	248.56	0.0017	4.988	H-1→L; H→L+1 (n–π*)
tg	-	169.69	0.7477	7.3066	H-3→L; H-2→L+1 (π–π*)
	239.2	233.33	0.0039	5.3137	H-1→ L+1; H→L+1 (n–π*)

[1] H – HOMO, L - LUMO

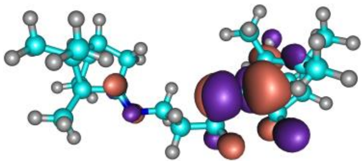

tg

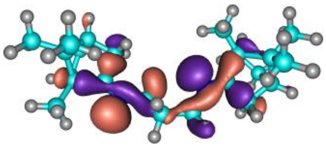

tg

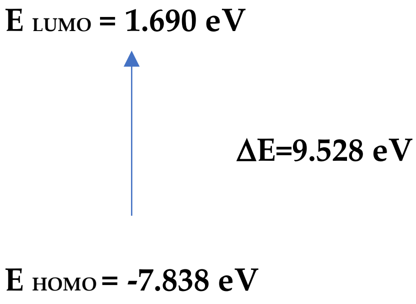

Cartesian coordinates of CPDA conformers tt-1 optimized CAM-B3LYP/6-31g(d,p) level of theory:

C	1.219165000000	-1.003858000000	-0.356414000000
H	0.966972000000	-1.659365000000	-1.200875000000
H	1.472981000000	-1.661226000000	0.488893000000
C	0.000743000000	-0.157262000000	-0.001804000000
H	-0.235007000000	0.497342000000	-0.846771000000
H	0.237159000000	0.497795000000	0.842631000000
C	-1.218552000000	-1.002271000000	0.353504000000
H	-1.473021000000	-1.660136000000	-0.491221000000
H	-0.967102000000	-1.657332000000	1.198548000000
N	2.316993000000	-0.151266000000	-0.774772000000
C	5.458918000000	0.565113000000	0.660402000000
C	5.468984000000	-0.980657000000	0.823016000000
C	4.675388000000	0.516407000000	-0.692163000000
C	6.112399000000	-1.457943000000	-0.493689000000
C	5.553950000000	-0.450017000000	-1.534697000000
C	3.975005000000	-1.339751000000	0.741211000000
C	3.471062000000	-0.325890000000	-0.289948000000
C	4.707145000000	1.303316000000	1.773799000000
C	6.855776000000	1.183836000000	0.562111000000
H	5.838918000000	-2.491819000000	-0.721049000000
H	7.203064000000	-1.423264000000	-0.443862000000
H	4.965145000000	-0.929191000000	-2.320964000000
H	6.354094000000	0.105819000000	-2.031145000000
H	4.729644000000	2.382539000000	1.597463000000
H	5.182746000000	1.121796000000	2.743030000000
H	3.657692000000	1.014611000000	1.854966000000
H	6.785834000000	2.255582000000	0.354072000000
H	7.384031000000	1.071614000000	1.514535000000
H	7.480264000000	0.739377000000	-0.213481000000
H	5.964151000000	-1.350162000000	1.725005000000
H	3.460384000000	-1.214115000000	1.698538000000
H	3.808453000000	-2.372998000000	0.421968000000
C	4.331042000000	1.819422000000	-1.378877000000

H	3.740713000000	1.628427000000	-2.277748000000
H	3.727235000000	2.461168000000	-0.732244000000
H	5.235662000000	2.364119000000	-1.665189000000
N	-2.315578000000	-0.148170000000	0.770951000000
C	-5.460540000000	0.562636000000	-0.660404000000
C	-5.470676000000	-0.983717000000	-0.817490000000
C	-4.674233000000	0.518881000000	0.690707000000
C	-6.111367000000	-1.456397000000	0.502200000000
C	-5.550915000000	-0.444693000000	1.538461000000
C	-3.976475000000	-1.342292000000	-0.737371000000
C	-3.470598000000	-0.324642000000	0.289060000000
C	-4.711216000000	1.297022000000	-1.777965000000
C	-6.857277000000	1.181516000000	-0.561408000000
H	-5.837315000000	-2.489428000000	0.732702000000
H	-7.202136000000	-1.422006000000	0.454471000000
H	-4.960419000000	-0.920970000000	2.325216000000
H	-6.350134000000	0.112792000000	2.034544000000
H	-4.732981000000	2.376806000000	-1.605015000000
H	-5.189212000000	1.112536000000	-2.745457000000
H	-3.662052000000	1.007739000000	-1.860766000000
H	-6.787016000000	2.253985000000	-0.357263000000
H	-7.387483000000	1.065914000000	-1.512341000000
H	-7.480100000000	0.739713000000	0.217031000000
H	-5.967594000000	-1.356510000000	-1.717156000000
H	-3.463750000000	-1.220108000000	-1.696164000000
H	-3.809099000000	-2.374334000000	-0.414672000000
C	-4.328622000000	1.824377000000	1.372075000000
H	-3.736096000000	1.636627000000	2.270180000000
H	-3.726500000000	2.463997000000	0.721768000000
H	-5.232661000000	2.369901000000	1.658655000000

Cartesian coordinates of CPDA conformers tt-2 optimized CAM-B3LYP/6-31g(d,p) level of theory:

C	-1.228284000000	-0.335241000000	1.189216000000
H	-1.492889000000	0.528455000000	1.818398000000
H	-0.989963000000	-1.168015000000	1.861759000000
C	0.000005000000	0.000003000000	0.341134000000
H	-0.218867000000	0.851335000000	-0.312059000000
H	0.218888000000	-0.851332000000	-0.312053000000
C	1.228278000000	0.335250000000	1.189237000000
H	0.989941000000	1.168020000000	1.861780000000
H	1.492881000000	-0.528446000000	1.818419000000
N	-2.321599000000	-0.773910000000	0.318859000000
C	-5.813478000000	-0.139547000000	0.207093000000
C	-5.302660000000	1.316230000000	0.454651000000
C	-4.566472000000	-0.531440000000	-0.665324000000
C	-5.084384000000	1.851307000000	-0.981551000000
C	-4.568040000000	0.595473000000	-1.746221000000
C	-3.899830000000	1.085108000000	1.059189000000
C	-3.428238000000	-0.145165000000	0.275621000000
C	-5.946469000000	-0.977400000000	1.486816000000
C	-7.153008000000	-0.204618000000	-0.536441000000
H	-4.361936000000	2.672674000000	-0.993068000000
H	-6.012154000000	2.235207000000	-1.413922000000
H	-3.567804000000	0.733743000000	-2.166049000000
H	-5.230201000000	0.321005000000	-2.573179000000
H	-6.298246000000	-1.985563000000	1.244729000000
H	-6.680168000000	-0.526170000000	2.164282000000
H	-5.004549000000	-1.085012000000	2.029662000000
H	-7.408445000000	-1.243425000000	-0.771027000000
H	-7.952636000000	0.195278000000	0.097534000000
H	-7.159610000000	0.357574000000	-1.471723000000
H	-5.955378000000	1.937462000000	1.072666000000
H	-3.938144000000	0.882854000000	2.134593000000
H	-3.240650000000	1.947841000000	0.916725000000

C	-4.480724000000	-1.939348000000	-1.215258000000
H	-3.528370000000	-2.083812000000	-1.731906000000
H	-4.523258000000	-2.680598000000	-0.411657000000
H	-5.296399000000	-2.138019000000	-1.918166000000
N	2.321604000000	0.773935000000	0.318901000000
C	5.813483000000	0.139546000000	0.207081000000
C	5.302651000000	-1.316222000000	0.454681000000
C	4.566463000000	0.531439000000	-0.665308000000
C	5.084380000000	-1.851315000000	-0.981513000000
C	4.568018000000	-0.595491000000	-1.746190000000
C	3.899812000000	-1.085109000000	1.059203000000
C	3.428242000000	0.145190000000	0.275666000000
C	5.946542000000	0.977437000000	1.486775000000
C	7.152982000000	0.204569000000	-0.536509000000
H	4.361943000000	-2.672692000000	-0.993022000000
H	6.012156000000	-2.235204000000	-1.413881000000
H	3.567776000000	-0.733770000000	-2.166001000000
H	5.230167000000	-0.321036000000	-2.573163000000
H	6.298337000000	1.985582000000	1.244641000000
H	6.680256000000	0.526209000000	2.164227000000
H	5.004648000000	1.085094000000	2.029654000000
H	7.408434000000	1.243365000000	-0.771129000000
H	7.952626000000	-0.195334000000	0.097439000000
H	7.159525000000	-0.357645000000	-1.471780000000
H	5.955368000000	-1.937448000000	1.072703000000
H	3.938113000000	-0.882888000000	2.134612000000
H	3.240629000000	-1.947834000000	0.916705000000
C	4.480720000000	1.939339000000	-1.215264000000
H	3.528375000000	2.083794000000	-1.731932000000
H	4.523237000000	2.680601000000	-0.411674000000
H	5.296407000000	2.138001000000	-1.918160000000

Cartesian coordinates of CPDA conformers gt optimized CAM-B3LYP/6-31g(d,p) level of theory:

C	-1.308609000000	-1.488624000000	-0.947283000000
H	-1.863570000000	-2.422830000000	-0.784726000000
H	-1.062994000000	-1.444409000000	-2.016622000000
C	-0.011451000000	-1.524770000000	-0.140723000000
H	0.533298000000	-2.444760000000	-0.381517000000
H	-0.242095000000	-1.567557000000	0.928791000000
C	0.890479000000	-0.322080000000	-0.396771000000
H	1.133671000000	-0.264941000000	-1.468963000000
H	0.332272000000	0.588320000000	-0.153004000000
N	-2.077216000000	-0.297794000000	-0.628770000000
C	-5.537290000000	0.323118000000	-0.338688000000
C	-5.468095000000	-1.014895000000	0.449432000000
C	-4.129737000000	0.797566000000	0.149885000000
C	-5.203427000000	-0.547523000000	1.893961000000
C	-4.270119000000	0.677633000000	1.693364000000
C	-4.158187000000	-1.641276000000	-0.059017000000
C	-3.275655000000	-0.402521000000	-0.241338000000
C	-5.626329000000	0.146635000000	-1.858554000000
C	-6.696439000000	1.227661000000	0.087008000000
H	-4.738197000000	-1.336801000000	2.490519000000
H	-6.129167000000	-0.270019000000	2.403101000000
H	-3.292986000000	0.552743000000	2.166696000000
H	-4.707589000000	1.592116000000	2.103329000000
H	-5.657310000000	1.121617000000	-2.353370000000
H	-6.543624000000	-0.385806000000	-2.129812000000
H	-4.782438000000	-0.400614000000	-2.282516000000
H	-6.645267000000	2.188359000000	-0.433861000000
H	-7.651582000000	0.763969000000	-0.180291000000
H	-6.720993000000	1.435391000000	1.157085000000
H	-6.340746000000	-1.664225000000	0.339535000000
H	-4.287922000000	-2.173133000000	-1.006006000000
H	-3.730529000000	-2.354101000000	0.652498000000

C	-3.602675000000	2.130098000000	-0.334460000000
H	-2.595101000000	2.300070000000	0.051388000000
H	-3.535669000000	2.158590000000	-1.425029000000
H	-4.244295000000	2.951672000000	-0.002591000000
N	2.068336000000	-0.392114000000	0.450137000000
C	5.520350000000	-0.259848000000	-0.269606000000
C	5.042600000000	0.921515000000	-1.160240000000
C	4.456479000000	0.019070000000	0.842344000000
C	5.134678000000	2.127493000000	-0.204954000000
C	4.713303000000	1.518592000000	1.160193000000
C	3.545500000000	0.619857000000	-1.347770000000
C	3.178230000000	0.024727000000	0.013048000000
C	5.367161000000	-1.635988000000	-0.927073000000
C	6.973383000000	-0.133037000000	0.195425000000
H	4.476696000000	2.940502000000	-0.523375000000
H	6.147231000000	2.535837000000	-0.168980000000
H	3.820022000000	1.987910000000	1.579965000000
H	5.504633000000	1.614914000000	1.908754000000
H	5.709726000000	-2.422528000000	-0.248491000000
H	5.978426000000	-1.698275000000	-1.833225000000
H	4.337274000000	-1.873972000000	-1.198983000000
H	7.224066000000	-0.938811000000	0.891763000000
H	7.650865000000	-0.220661000000	-0.660251000000
H	7.194691000000	0.811362000000	0.693558000000
H	5.590797000000	1.047948000000	-2.097711000000
H	3.358979000000	-0.097918000000	-2.152641000000
H	2.959238000000	1.514800000000	-1.577985000000
C	4.415376000000	-0.886469000000	2.053275000000
H	3.581432000000	-0.609584000000	2.701943000000
H	4.260666000000	-1.928992000000	1.763466000000
H	5.343081000000	-0.818442000000	2.629223000000

Cartesian coordinates of CPDA conformers tg optimized CAM-B3LYP/6-31g(d,p) level of theory:

C	-0.975556000000	-1.514871000000	-0.544730000000
H	-0.806302000000	-2.134045000000	-1.436207000000
H	-0.614061000000	-0.505707000000	-0.777068000000
C	-0.153884000000	-2.082845000000	0.608543000000
H	-0.489770000000	-3.103480000000	0.818541000000
H	-0.345438000000	-1.490366000000	1.509889000000
C	1.341583000000	-2.075653000000	0.321731000000
H	1.873418000000	-2.596988000000	1.130486000000
H	1.543233000000	-2.647713000000	-0.596663000000
N	-2.392221000000	-1.577723000000	-0.228238000000
C	-4.730643000000	0.916474000000	0.631042000000
C	-4.062068000000	1.626136000000	-0.579864000000
C	-4.588193000000	-0.507728000000	0.000645000000
C	-4.922153000000	1.154247000000	-1.768521000000
C	-5.263099000000	-0.311719000000	-1.385467000000
C	-2.710765000000	0.899763000000	-0.695888000000
C	-3.095691000000	-0.530562000000	-0.306756000000
C	-3.964187000000	1.079595000000	1.948514000000
C	-6.174778000000	1.356852000000	0.883812000000
H	-4.373902000000	1.225442000000	-2.711805000000
H	-5.823204000000	1.761831000000	-1.879775000000
H	-4.888960000000	-1.040227000000	-2.109256000000
H	-6.342294000000	-0.469260000000	-1.304747000000
H	-4.474497000000	0.543859000000	2.754259000000
H	-3.918554000000	2.134877000000	2.236564000000
H	-2.942357000000	0.698525000000	1.907135000000
H	-6.621358000000	0.762423000000	1.686410000000
H	-6.197725000000	2.403813000000	1.203399000000
H	-6.820487000000	1.265853000000	0.010024000000
H	-3.975062000000	2.712460000000	-0.491716000000
H	-1.955278000000	1.300876000000	-0.014007000000
H	-2.290628000000	0.955194000000	-1.704590000000

C	-5.085846000000	-1.702083000000	0.784124000000
H	-4.857132000000	-2.626006000000	0.248449000000
H	-4.594280000000	-1.769095000000	1.758028000000
H	-6.167224000000	-1.648495000000	0.941901000000
N	1.836952000000	-0.710947000000	0.179896000000
C	5.091533000000	0.595658000000	0.565317000000
C	5.432320000000	-0.670698000000	-0.271087000000
C	3.725579000000	0.829325000000	-0.160598000000
C	5.337643000000	-0.156255000000	-1.721269000000
C	4.159586000000	0.853431000000	-1.653251000000
C	4.203034000000	-1.565902000000	-0.041267000000
C	3.078727000000	-0.537103000000	0.018230000000
C	4.944063000000	0.329269000000	2.067634000000
C	6.102091000000	1.733398000000	0.398733000000
H	5.150672000000	-0.973256000000	-2.423309000000
H	6.264545000000	0.326802000000	-2.038352000000
H	3.327004000000	0.583782000000	-2.307951000000
H	4.473123000000	1.861942000000	-1.936729000000
H	4.694773000000	1.254425000000	2.595118000000
H	5.885622000000	-0.040732000000	2.485882000000
H	4.163000000000	-0.394975000000	2.306069000000
H	5.762907000000	2.628082000000	0.929122000000
H	7.065630000000	1.445429000000	0.831513000000
H	6.280456000000	2.014885000000	-0.639459000000
H	6.384137000000	-1.148259000000	-0.024290000000
H	4.257319000000	-2.133443000000	0.893630000000
H	4.048298000000	-2.295640000000	-0.843227000000
C	2.862813000000	1.995516000000	0.267579000000
H	1.922563000000	1.993039000000	-0.288498000000
H	2.606327000000	1.931105000000	1.328099000000
H	3.369707000000	2.948253000000	0.088295000000

Cartesian coordinates of Dimer 1 optimized CAM-B3LYP/6-31g(d,p) level of theory:

C	-0.950597000000	-1.630216000000	-0.116181000000
H	-0.957429000000	-0.620382000000	-0.541849000000
H	-1.721067000000	-2.211794000000	-0.634441000000
C	-1.304054000000	-1.522484000000	1.366655000000
H	-0.442536000000	-1.107915000000	1.900874000000
H	-1.481842000000	-2.523934000000	1.776644000000
C	-2.515504000000	-0.633510000000	1.613066000000
H	-2.705535000000	-0.557434000000	2.693907000000
H	-2.275404000000	0.377888000000	1.258466000000
N	0.395150000000	-2.161952000000	-0.269773000000
C	1.605778000000	-5.396165000000	-0.957131000000
C	0.497554000000	-5.326283000000	-2.045407000000
C	1.955135000000	-3.876338000000	-1.077992000000
C	1.248272000000	-4.741717000000	-3.257982000000
C	2.226097000000	-3.730226000000	-2.600976000000
C	-0.427523000000	-4.214387000000	-1.519738000000
C	0.577708000000	-3.256113000000	-0.874777000000
C	1.091026000000	-5.795257000000	0.430332000000
C	2.756717000000	-6.342386000000	-1.309044000000
H	0.561007000000	-4.264663000000	-3.961747000000
H	1.782549000000	-5.516347000000	-3.812889000000
H	2.060290000000	-2.701119000000	-2.929829000000
H	3.269357000000	-3.971113000000	-2.823775000000
H	1.913457000000	-5.810060000000	1.151589000000
H	0.662462000000	-6.802344000000	0.404587000000
H	0.331865000000	-5.116899000000	0.823320000000
H	3.543532000000	-6.287750000000	-0.550774000000
H	2.398719000000	-7.376867000000	-1.331095000000
H	3.217152000000	-6.134295000000	-2.275409000000
H	-0.014881000000	-6.270762000000	-2.247301000000
H	-1.149758000000	-4.577665000000	-0.783169000000
H	-1.001954000000	-3.735452000000	-2.318130000000
C	3.037166000000	-3.305792000000	-0.188055000000

H	3.131465000000	-2.230181000000	-0.358347000000
H	2.793814000000	-3.442075000000	0.868958000000
H	4.004033000000	-3.777578000000	-0.388675000000
N	-3.688931000000	-1.135132000000	0.906842000000
C	-7.128472000000	-0.875127000000	1.658219000000
C	-6.697922000000	0.588276000000	1.961060000000
C	-6.110020000000	-1.036293000000	0.482407000000
C	-6.882656000000	1.285887000000	0.598576000000
C	-6.461799000000	0.184871000000	-0.412860000000
C	-5.181873000000	0.454336000000	2.184874000000
C	-4.816140000000	-0.630297000000	1.175507000000
C	-6.880440000000	-1.848995000000	2.815688000000
C	-8.597594000000	-1.011434000000	1.250497000000
H	-6.262334000000	2.183077000000	0.522725000000
H	-7.917343000000	1.600848000000	0.444206000000
H	-5.608464000000	0.477154000000	-1.030115000000
H	-7.276643000000	-0.071367000000	-1.095850000000
H	-7.200621000000	-2.857967000000	2.539871000000
H	-7.458964000000	-1.553071000000	3.696787000000
H	-5.831143000000	-1.915037000000	3.108579000000
H	-8.820743000000	-2.042197000000	0.959559000000
H	-9.246469000000	-0.765518000000	2.097442000000
H	-8.884208000000	-0.366216000000	0.419590000000
H	-7.230156000000	1.067565000000	2.787091000000
H	-4.928402000000	0.138450000000	3.202253000000
H	-4.636465000000	1.385181000000	1.998437000000
C	-6.043233000000	-2.359151000000	-0.247976000000
H	-5.244853000000	-2.337233000000	-0.993157000000
H	-5.816546000000	-3.179286000000	0.438132000000
H	-6.987141000000	-2.579600000000	-0.755430000000
C	1.004781000000	1.470677000000	0.250484000000
H	1.089394000000	0.391462000000	0.072617000000
H	1.792695000000	1.964375000000	-0.331333000000
C	1.239909000000	1.744559000000	1.734036000000
H	0.423839000000	1.299223000000	2.312934000000

H	1.213959000000	2.825023000000	1.914811000000
C	2.572478000000	1.187362000000	2.220096000000
H	2.671177000000	1.383199000000	3.296114000000
H	2.578625000000	0.095081000000	2.092354000000
N	-0.340128000000	1.878039000000	-0.129600000000
C	-1.582822000000	4.841620000000	-1.566959000000
C	-0.364679000000	4.622276000000	-2.507164000000
C	-1.867909000000	3.310295000000	-1.418703000000
C	-0.962211000000	3.776811000000	-3.648386000000
C	-1.968410000000	2.858821000000	-2.902831000000
C	0.534833000000	3.682074000000	-1.686104000000
C	-0.500240000000	2.816858000000	-0.961433000000
C	-1.229963000000	5.528862000000	-0.243095000000
C	-2.720537000000	5.639293000000	-2.209228000000
H	-0.188436000000	3.211617000000	-4.174689000000
H	-1.461520000000	4.400341000000	-4.393632000000
H	-1.734094000000	1.796095000000	-3.004412000000
H	-2.990089000000	2.995195000000	-3.268140000000
H	-2.122669000000	5.636871000000	0.380044000000
H	-0.837759000000	6.534176000000	-0.427197000000
H	-0.490998000000	4.983305000000	0.346207000000
H	-3.581834000000	5.689366000000	-1.536462000000
H	-2.397047000000	6.667967000000	-2.398765000000
H	-3.067210000000	5.224880000000	-3.156346000000
H	0.135887000000	5.535762000000	-2.839255000000
H	1.165790000000	4.216542000000	-0.970210000000
H	1.202724000000	3.087039000000	-2.315962000000
C	-3.027397000000	2.870992000000	-0.551459000000
H	-3.110270000000	1.781126000000	-0.547761000000
H	-2.889400000000	3.189290000000	0.485356000000
H	-3.970794000000	3.285121000000	-0.921265000000
N	3.682491000000	1.834223000000	1.533991000000
C	7.136206000000	1.129530000000	1.481951000000
C	6.594364000000	-0.316221000000	1.298137000000
C	5.976121000000	1.775085000000	0.655620000000

C	6.492299000000	-0.449739000000	-0.234237000000
C	6.044841000000	0.971147000000	-0.673268000000
C	5.153428000000	-0.208792000000	1.826311000000
C	4.764000000000	1.198892000000	1.376298000000
C	7.177327000000	1.597736000000	2.940953000000
C	8.532153000000	1.343857000000	0.891857000000
H	5.774821000000	-1.223210000000	-0.520978000000
H	7.451041000000	-0.725610000000	-0.679162000000
H	5.077655000000	0.974912000000	-1.182400000000
H	6.765508000000	1.431446000000	-1.354969000000
H	7.556757000000	2.621836000000	3.002161000000
H	7.851263000000	0.965093000000	3.527605000000
H	6.200131000000	1.588935000000	3.427294000000
H	8.823615000000	2.395589000000	0.968434000000
H	9.270257000000	0.760759000000	1.452195000000
H	8.614570000000	1.056136000000	-0.156719000000
H	7.186611000000	-1.099208000000	1.779211000000
H	5.093088000000	-0.297195000000	2.915470000000
H	4.491497000000	-0.974388000000	1.409377000000
C	5.952471000000	3.277710000000	0.485220000000
H	5.056522000000	3.581136000000	-0.061069000000
H	5.922903000000	3.786577000000	1.452054000000
H	6.830896000000	3.626431000000	-0.065897000000

Cartesian coordinates of Dimer 2 optimized CAM-B3LYP/6-31g(d,p) level of theory:

C	1.529835000000	1.040739000000	-0.335821000000
H	1.024226000000	0.166474000000	0.091989000000
H	1.452367000000	1.855574000000	0.394025000000
C	0.803800000000	1.458819000000	-1.613546000000
H	0.809434000000	0.626096000000	-2.324750000000
H	1.349575000000	2.285572000000	-2.081529000000
C	-0.626427000000	1.910735000000	-1.347161000000
H	-1.119118000000	2.131272000000	-2.303576000000
H	-1.197920000000	1.091221000000	-0.887463000000
N	2.904367000000	0.662146000000	-0.632548000000
C	5.745481000000	2.673457000000	-1.162852000000
C	5.217864000000	3.353631000000	0.131367000000
C	5.295835000000	1.242861000000	-0.712129000000
C	5.939449000000	2.571090000000	1.245588000000
C	5.970310000000	1.123981000000	0.683454000000
C	3.743550000000	2.916299000000	0.168125000000
C	3.826190000000	1.500794000000	-0.406822000000
C	5.056834000000	3.155439000000	-2.444618000000
C	7.254573000000	2.828583000000	-1.367989000000
H	5.402484000000	2.641269000000	2.195306000000
H	6.946307000000	2.956314000000	1.421991000000
H	5.440418000000	0.408219000000	1.317192000000
H	6.993168000000	0.752847000000	0.574581000000
H	5.452843000000	2.622081000000	-3.313689000000
H	5.246556000000	4.221969000000	-2.602360000000
H	3.975899000000	3.004992000000	-2.441195000000
H	7.582110000000	2.269727000000	-2.249569000000
H	7.502031000000	3.880963000000	-1.540831000000
H	7.850853000000	2.486013000000	-0.521857000000
H	5.359907000000	4.436547000000	0.178128000000
H	3.092094000000	3.549017000000	-0.441376000000
H	3.330023000000	2.923229000000	1.181367000000

C	5.557957000000	0.078627000000	-1.641821000000
H	5.198029000000	-0.853154000000	-1.199382000000
H	5.035743000000	0.204946000000	-2.593399000000
H	6.628111000000	-0.029096000000	-1.842113000000
N	-0.636380000000	3.109653000000	-0.518270000000
C	-3.315059000000	5.082004000000	0.652428000000
C	-3.895898000000	3.673642000000	0.966279000000
C	-1.843496000000	4.654314000000	0.967261000000
C	-3.395857000000	3.411440000000	2.400600000000
C	-1.984012000000	4.058845000000	2.396540000000
C	-3.077536000000	2.757882000000	0.040392000000
C	-1.709303000000	3.433826000000	0.067147000000
C	-3.521609000000	5.529996000000	-0.798635000000
C	-3.861630000000	6.189219000000	1.557074000000
H	-3.366891000000	2.341694000000	2.625337000000
H	-4.048562000000	3.873121000000	3.144822000000
H	-1.184774000000	3.342311000000	2.602768000000
H	-1.899909000000	4.852464000000	3.144125000000
H	-3.082443000000	6.518975000000	-0.958422000000
H	-4.589275000000	5.606753000000	-1.027673000000
H	-3.069413000000	4.857099000000	-1.529444000000
H	-3.355887000000	7.137195000000	1.351083000000
H	-4.928048000000	6.341154000000	1.361483000000
H	-3.749719000000	5.982040000000	2.621681000000
H	-4.977756000000	3.578091000000	0.842539000000
H	-3.469933000000	2.722166000000	-0.981045000000
H	-3.032823000000	1.722811000000	0.395892000000
C	-0.739046000000	5.676013000000	0.812446000000
H	0.231757000000	5.215189000000	1.008933000000
H	-0.704210000000	6.071079000000	-0.206000000000
H	-0.873759000000	6.510845000000	1.506380000000
C	-1.625043000000	-2.053410000000	-1.431081000000
H	-1.097325000000	-1.118702000000	-1.662774000000
H	-1.456764000000	-2.266907000000	-0.367810000000
C	-1.008580000000	-3.173706000000	-2.262678000000

H	-1.137281000000	-2.944992000000	-3.325511000000
H	-1.546685000000	-4.106434000000	-2.063117000000
C	0.467449000000	-3.377843000000	-1.943475000000
H	0.879618000000	-4.140237000000	-2.618207000000
H	1.024066000000	-2.449482000000	-2.142806000000
N	-3.023198000000	-1.868228000000	-1.779505000000
C	-5.998923000000	-2.722348000000	-0.095037000000
C	-5.231932000000	-2.077244000000	1.093360000000
C	-5.391430000000	-1.739313000000	-1.148990000000
C	-5.647498000000	-0.595554000000	1.002180000000
C	-5.736139000000	-0.356416000000	-0.529897000000
C	-3.766142000000	-2.155545000000	0.633797000000
C	-3.903017000000	-1.918054000000	-0.873270000000
C	-5.633408000000	-4.189139000000	-0.349284000000
C	-7.521879000000	-2.646248000000	0.040885000000
H	-4.914894000000	0.055587000000	1.488164000000
H	-6.605675000000	-0.414053000000	1.494454000000
H	-5.044919000000	0.413402000000	-0.883215000000
H	-6.739582000000	-0.045688000000	-0.834231000000
H	-6.194523000000	-4.577987000000	-1.203930000000
H	-5.892671000000	-4.804754000000	0.518064000000
H	-4.574555000000	-4.342272000000	-0.564732000000
H	-8.006956000000	-3.030586000000	-0.861257000000
H	-7.855362000000	-3.267415000000	0.878539000000
H	-7.898667000000	-1.637110000000	0.210859000000
H	-5.415728000000	-2.535621000000	2.068752000000
H	-3.311371000000	-3.129331000000	0.836819000000
H	-3.134441000000	-1.403870000000	1.117065000000
C	-5.784135000000	-1.898686000000	-2.601045000000
H	-5.238032000000	-1.183954000000	-3.220619000000
H	-5.533921000000	-2.896027000000	-2.971277000000
H	-6.856638000000	-1.733332000000	-2.740230000000
N	0.632310000000	-3.832430000000	-0.569044000000
C	3.519303000000	-4.587646000000	1.291364000000
C	3.985346000000	-3.239430000000	0.673233000000

C	2.031631000000	-4.122130000000	1.427609000000
C	3.530808000000	-2.205705000000	1.723251000000
C	2.184232000000	-2.796393000000	2.223743000000
C	3.046776000000	-3.070946000000	-0.535549000000
C	1.751665000000	-3.674924000000	-0.001221000000
C	3.695932000000	-5.793485000000	0.362206000000
C	4.202945000000	-4.925790000000	2.618542000000
H	3.413109000000	-1.217235000000	1.273637000000
H	4.254407000000	-2.111321000000	2.536993000000
H	1.334564000000	-2.132337000000	2.043300000000
H	2.203417000000	-3.002794000000	3.297739000000
H	3.332819000000	-6.704820000000	0.846289000000
H	4.754881000000	-5.947190000000	0.130725000000
H	3.157125000000	-5.694669000000	-0.581865000000
H	3.774254000000	-5.834475000000	3.051991000000
H	5.268424000000	-5.116695000000	2.453292000000
H	4.124110000000	-4.135903000000	3.366191000000
H	5.048316000000	-3.186837000000	0.421890000000
H	3.389522000000	-3.619718000000	-1.418930000000
H	2.929793000000	-2.022024000000	-0.830364000000
C	1.015815000000	-5.088348000000	1.995596000000
H	0.019055000000	-4.641479000000	1.978163000000
H	0.963231000000	-6.004387000000	1.401718000000
H	1.258602000000	-5.355022000000	3.028571000000
